# Supplementary material for: Continuous cholinergic-dopaminergic updating in the nucleus accumbens underlies approaches to reward-predicting cues
Source: Nat Commun. 2022 Dec 24;13:7924. doi: 10.1038/s41467-022-35601-x (PMC9789106; doi:10.1038/s41467-022-35601-x)
Supplement: Supplementary file 1 — Supplementary Information [file 41467_2022_35601_MOESM1_ESM.pdf]

Title: Continuous Cholinergic-Dopaminergic Updating in the Nucleus Accumbens Underlies Approaches to Reward-Predicting Cues

Author List

Miguel Skirzewski[#], Oren Princz-Lebel, Liliana German-Castelan, Alycia M. Crooks, Gerard Kyungwook Kim, Sophie Henke Tarnow, Amy Reichelt, Sara Memar, Daniel Palmer, Yulong Li, R. Jane Rylett, Lisa M. Saksida, Vania F. Prado, Marco A.M. Prado[#], Timothy J. Bussey[#]

[#] Correspondence to Miguel Skirzewski, mskirzew@uwo.ca, Marco A.M. Prado, mprado@uwo.ca, and Timothy J. Bussey, tbussey@uwo.ca. Robarts Research Institute, Department of Physiology and Pharmacology, and Department of Anatomy and Cell Biology, Western University, 1151 Richmond St. N, N6A 5B7, London, Ontario, Canada.

Supplementary Figures

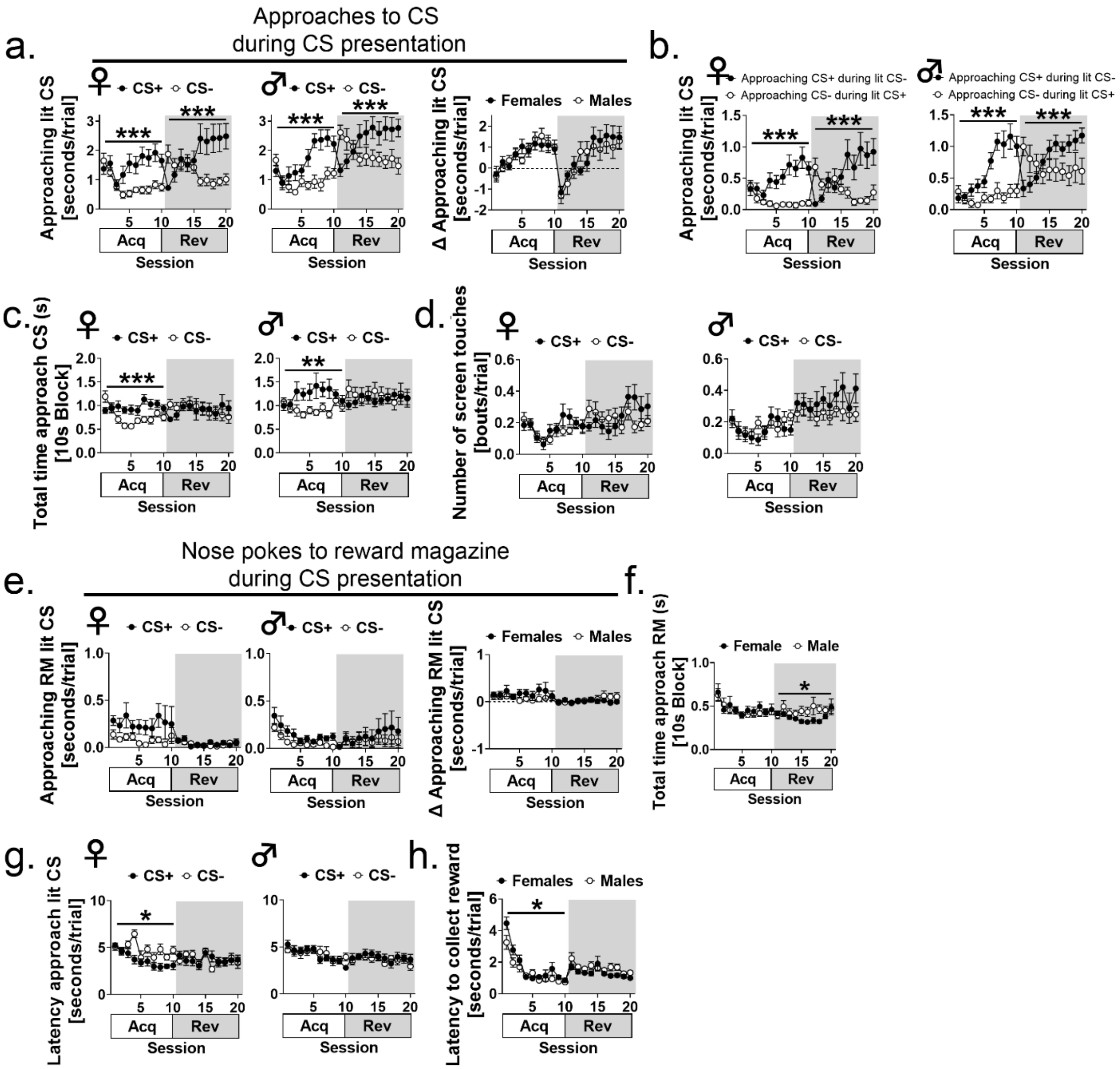

Supplementary Figure 1: C57BL/6j mice performing an automated touchscreen Autoshaping task anticipate rewards by approaching a reward-predicting stimulus.

(a) Female and male C57BL/6j mice spent more time approaching the CS+ (filled circles) than the CS- (blank circles) during acquisition (Acq, S1→S10) and reversal (Rev, S11→S20) sessions (two-way RM-ANOVA SessionXCS interaction, Acq-females: $F(9,198)=4.809$, $p<0.0001$, $\eta_p^2=0.41$ [0.28, 0.53]; Rev-females: $F(9,198)=14.12$, $p<0.0001$, $\eta_p^2=0.75$ [0.62, 0.89]; Acq-males: $F(9,198)=6.378$, $p<0.0001$, $\eta_p^2=0.48$ [0.35, 0.61]; Rev-males: $F(9,198)=10.42$, $p<0.0001$, $\eta_p^2=0.64$ [0.51, 0.77]). No differences

were observed when comparing the relative time (Δ [CS+ − CS-]) female and male mice approached the CS ($p>0.05$). (b) During the CS+ presentation (blank circles), both female and male mice rarely explored the opposite CS- screen. Moreover, mice spent more time visiting the CS+ during the CS- presentation (filled circles) (two-way RM-ANOVA SessionXCS interaction, Acq-females: $F(9,198)=4.809$, $p<0.0001$; Rev-females: $F(9,198)=12.17$, $p<0.0001$; Acq-males: $F(9,198)=6.734$, $p<0.0001$; Rev-males: $F(9,198)=9.021$, $p<0.0001$). No sex differences were observed ($p>0.05$). (c) When recording the total time mice approached both CS screens (10s blocks) during no stimulus presentation (e.g. ITI), both female and male mice spent more time visiting the CS+ than CS- during acquisition training sessions (two-way RM-ANOVA SessionXCS interaction, females: $F(9,198)=6.125$, $p<0.0001$; males: $F(9,198)=2.992$, $p=0.0023$), but not during reversal sessions ($p>0.05$). (d) Female and male mice did not show differences at touching the CS during presentation ($p>0.05$). (e) Approaches to the reward magazine (RM) during CS presentation. No sex differences were observed ($p>0.05$). (f) Female and male mice similarly visited the RM during no CS presentation ($p>0.05$). (g) Females, but not male mice ($p>0.05$), were slower to approach the CS- during acquisition sessions (two-way RM-ANOVA SessionXCS interaction, Acq: $F(9,198)=2.430$, $p=0.0122$; Rev: $p>0.05$). (h) Female mice were slower than males to collect rewards during acquisition sessions (two-way RM-ANOVA SessionXSex interaction, $F(9,198)=1.989$, $p=0.0423$). No differences were observed during reversal sessions ($p>0.05$). A total of N=12 female and N=12 male mice were used. Post-hoc Tukey's test: ***$p<0.0001$, **$p<0.001$, *$p<0.05$. No adjustments were made for multiple comparison analyses. Data are presented as the mean ± SEM. Source data are provided as a Source Data file.

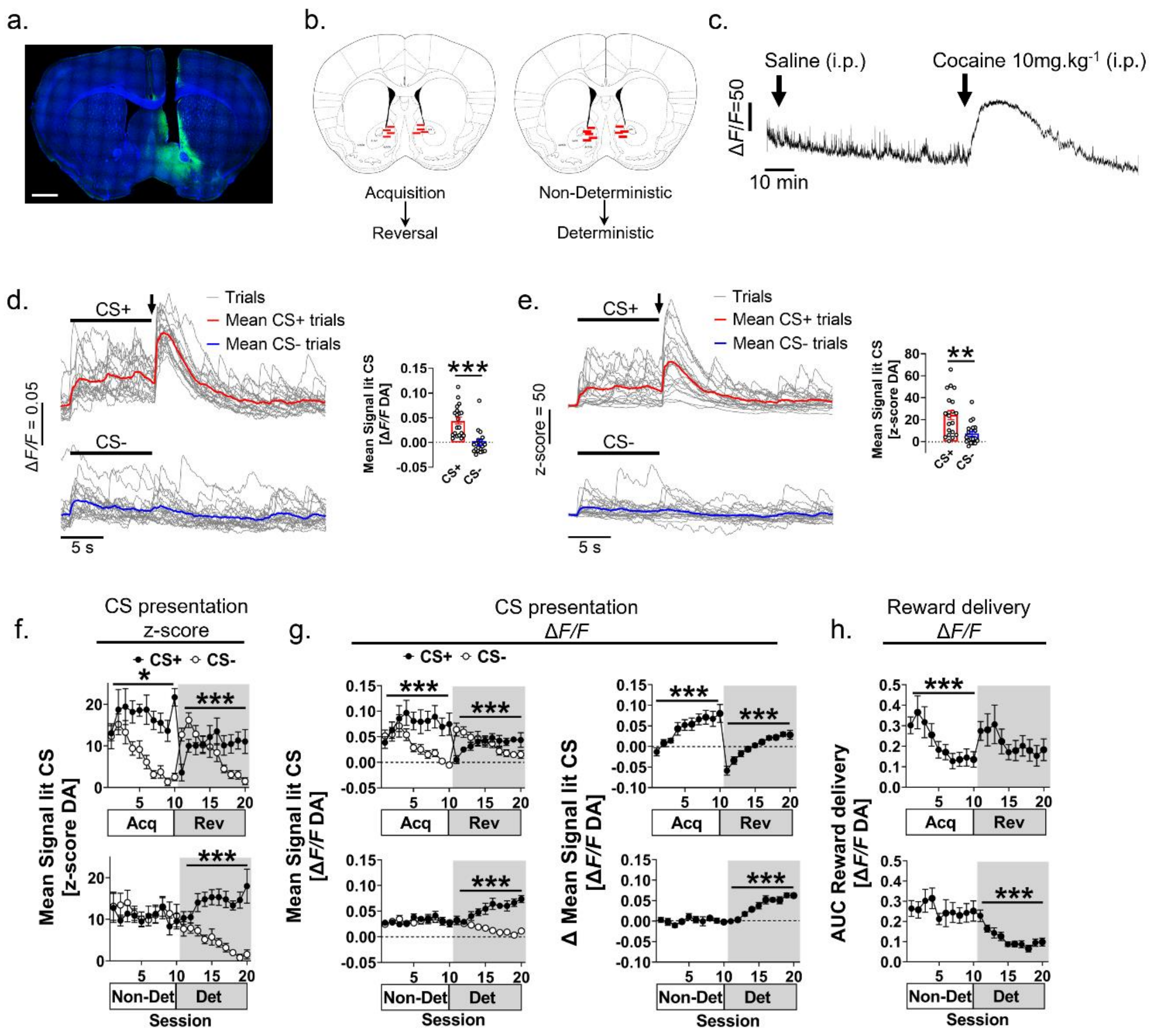

Supplementary Figure 2: in vivo nucleus accumbens dopamine recordings using fibre photometry.

(a) Representative coronal brain section showing the probe track lesion and GFP immunoreactivity ($GRAB_{DA2m}$, green) within the nucleus accumbens. Nuclei were stained with Hoechst (blue). Scale bar-1mm. (b) Depicted brain sections illustrating the tip (red) of each fibre optic in the nucleus accumbens of mice performing Acquisition (Acq) and reversal (Rev) sessions, or non-deterministic (Non-Det) and deterministic (Det) sessions. (c) Representative nucleus accumbens dopamine dynamics (ΔF/F). After 5min of habituation, the mouse received a single saline injection (i.p.) and dopamine recorded for 1h. Then, after an injection of cocaine (10mg.kg^{-1}, i.p.), dopamine was recorded for an additional 1h. Arrow bars indicate time of saline and cocaine injections, respectively. (d) (left) Representative trial-by-trial (gray traces) and

trial average (CS+, red; CS- blue) dopamine dynamics (ΔF/F) from a single mouse during the last acquisition session (S10). Bar represents CS presentation (10s), arrow bar indicates reward delivery. (right) Mean DA signal during CS presentation from individual trials (blank circles). Data shows the amplitude of the DA signal during CS+ trials was larger than during CS- trials (two-tailed t-test, $t(19)=5.896$, ***$p<0.0001$). (e) (left) Representative trial-by-trial (gray traces) and trial average (CS+, red; CS- blue) dopamine dynamics (z-score) from similar recordings as in (d) during the last acquisition session (S10). Bar represents CS presentation (10s), arrow bar reward delivery. (right) Mean DA signal during CS presentation from individual trials (blank circles). Data shows the amplitude of the DA signal during CS+ trials was larger than during CS- trials (two-tailed t-test, $t(19)=3.818$, **$p=0.0012$). (f) (top) Mean DA dynamics during CS+ (filled circles) and CS- (blank circles) across acquisition (Acq) and reversal (Rev) sessions (two-way RM-ANOVA SessionXCS interaction, Acq: $F(9,126)=2.537$, $p=0.0104$; Rev: $F(9,126)=6.873$, $p<0.0001$). (bottom) Mean DA dynamics during CS+ (filled circles) and CS- (blank circles) across non-deterministic (Non-Det) and deterministic (Det) sessions (two-way RM-ANOVA SessionXCS interaction, Non-Det: $p>0.05$; Det: $F(9,144)=6.150$, $p<0.0001$). (g) (left panels) Mean DA signal (ΔF/F) during CS presentation (10s) in acquisition and reversal sessions (two-way RM-ANOVA SessionXCS, Acq: $F(9,126)=5.829$, $p<0.0001$; Rev: $F(9,126)=12.75$, $p<0.0001$). Mean DA signal (ΔF/F) during CS presentation (10s) in non-deterministic and deterministic sessions (two-way RM-ANOVA SessionXCS, Non-Det: $p>0.05$; Det: $F(9,144)=16.48$, $p<0.0001$). (right panels) Relative increase of DA signal (Δ) during CS stimuli across sessions (one-way RM-ANOVA, Acq: $F(7,63)=17.08$, $p<0.0001$; Rev: $F(7,63)=14.27$, $p<0.0001$; Non-Det: $p>0.05$; Det: $F(8,72)=7.425$, $p<0.0001$). (h) During the reward delivery, the amplitude of the DA response (AUC, ΔF/F) significantly reduced across the acquisition, reversal and deterministic training sessions (one-way RM-ANOVA, Acq: $F(7,63)=30.81$, $p<0.0001$; Rev: $F(7,63)=22.63$, $p<0.0001$; Non-Det: $p>0.05$; Det: $F(8,72)=7.406$, $p<0.0001$). At least otherwise indicated, a total of total of N=8 mice (n=4♂, n=4♀) were used for acquisition and reversal training sessions, and N=9 mice (n=5♂, n=4♀) used for non-deterministic and deterministic sessions. Post-hoc Tukey's test: ***$p<0.0001$, **$p<0.001$,

*p<0.05. No adjustments were made for multiple comparison analyses. Data are presented as the mean ± SEM. Source data are provided as a Source Data file.

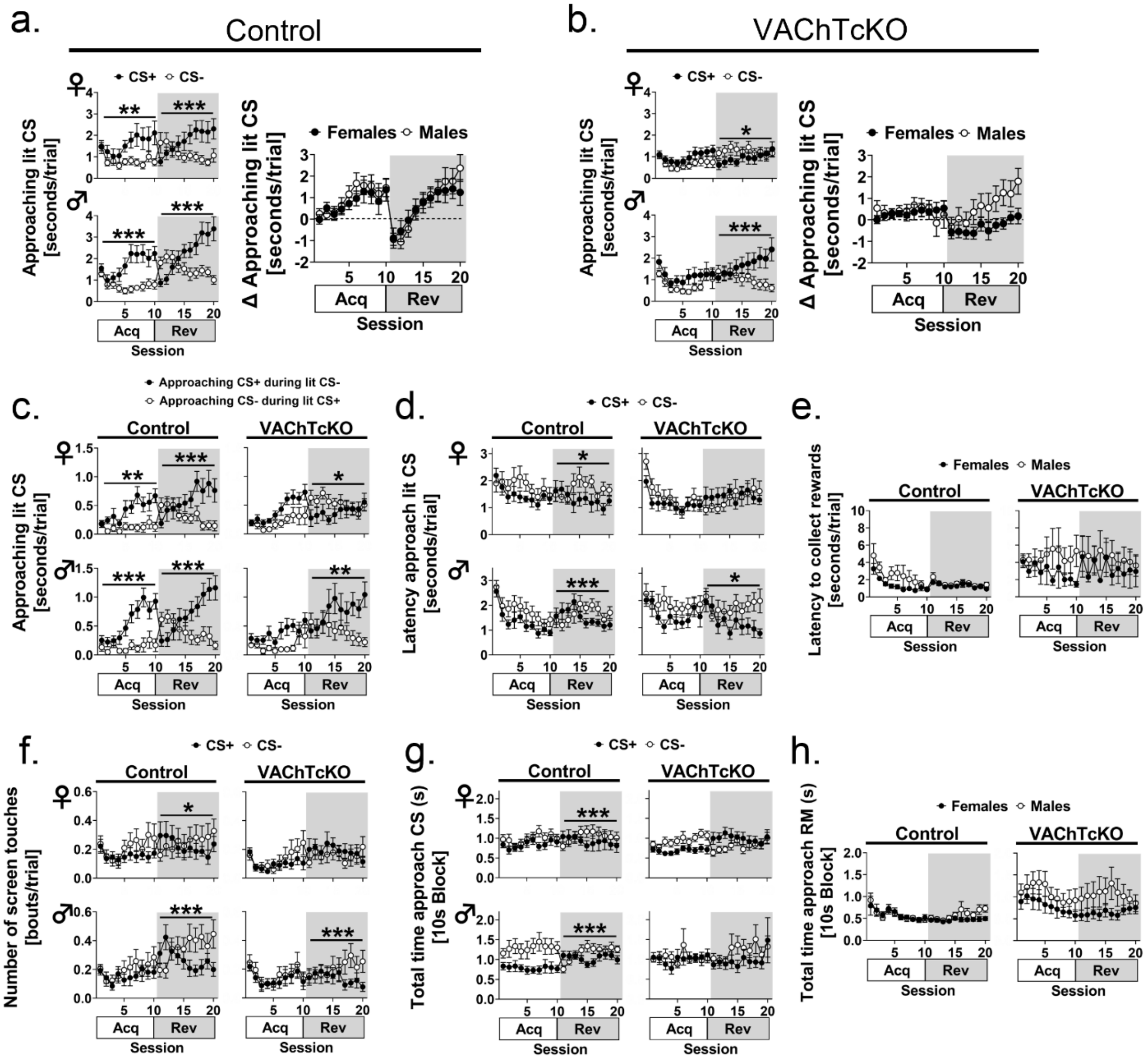

Supplementary Figure 3: Conditional VAChTcKO mice are impaired to approach reward-predicting conditioned stimuli.

(a) (left panels) Both male and female control littermate mice similarly spent more time approaching the CS+ (filled circles) than the CS- (blank circles) during presentation across acquisition (Acq, S1→S10) and reversal (Rev, S11→S20) sessions (two-way RM-ANOVA SessionXCS interaction, Acq-females: $F(9,180)=2.588$, $p=0.0079$; Rev-females: $F(9,180)=5.707$, $p<0.0001$; Acq-males: $F(9,216)=6.415$, $p<0.0001$; Rev-males: $F(9,216)=12.91$, $p<0.0001$). (right panel) The relative time mice spent visiting both CS (Δ) revealed that males (blank circles) and females (filled circles) similarly spent more time approaching the CS+ than the CS- ($p>0.05$). (b) (left panels) In contrast, male and

female VAChTcKO mice spent similar time approaching the CS+ and the CS- across sessions ($p>0.05$). Significantly, only after the S15-17 training sessions, VAChTcKO mice visited more the CS+ than the CS- (two-way RM-ANOVA SessionXCS interaction, Rev-females: $F(9,234)=1.992$, $p=0.0410$; Rev-males: $F(9,180)=5.924$, $p<0.0001$). (right panel) No sex differences were observed when comparing the relative Δ time mice approached the CS across sessions ($p>0.05$). (c) During CS+ presentation (blank circles), male and female mice from both genotypes rarely explored the CS-. Instead, mice visited the CS+ screen when the CS- was presented (filled circles) (two-way RM-ANOVA SessionXCS interaction, Acq-Control females: $F(9,180)=3.007$, $p=0.0023$; Rev-Control females: $F(9,180)=5.274$, $p<0.0001$; Acq-Control males: $F(9,216)=6.069$, $p<0.0001$; Rev-Control males: $F(9,216)=10.09$, $p<0.0001$; Acq-VAChTcKO females: $p>0.05$; Rev-VAChTcKO females: $F(9,234)=2.461$, $p=0.0107$; Acq-VAChTcKO males: $p>0.05$; Rev-VAChTcKO males: $F(9,180)=3.080$, $p=0.0018$). (d) Although no sex or genotype differences were observed between mice ($p>0.05$), latency time to approach CS- was larger in both genotypes during reversal sessions (Mixed-effects Model SessionXCS interaction, Acq-Control females: $p>0.05$; Rev-Control females: $F(9,171)=1.970$, $p<0.05$; Acq-Control males: $p>0.05$; Rev-Control males: $F(9,201)=3.397$, $p<0.05$; Acq-VAChTcKO females: $p>0.05$; Rev-VAChTcKO females: $p>0.05$; Acq-VAChTcKO males: $p>0.05$; Rev-VAChTcKO males: $F(9,164)=1.951$, $p<0.05$). (e) Latency time to collect rewards was similar across sex and between genotypes ($p>0.05$). (f) During acquisition sessions, no differences at touching both CS+/CS- screens during presentation were observed in both genotypes ($p>0.05$). However, both control (two-way RM-ANOVA SessionXCS interaction, Acq-females: $p>0.05$; Rev-females: $F(9,180)=2.282$, $p=0.0191$; Acq-males: $p>0.05$; Rev-males: $F(9,216)=7.381$, $p<0.0001$) and VAChTcKO mice (two-way RM-ANOVA SessionXCS interaction, Acq-females: $p>0.05$; Rev-females: $p>0.05$; Acq-males: $p>0.05$; Rev-males: $F(9,180)=4.084$, $p<0.0001$) touched the CS- more often during reversal training sessions. (g) No differences between sex and across genotypes were observed when recording the total time mice approached both CS screens ($p>0.05$). However, control littermate mice spent more time visiting the CS- over CS+ (Mixed-effects Model SessionXCS interaction, Acq-Control females: $p>0.05$; Rev-Control females:

F(9,178)=3.411, p<0.05; Acq-Control males: p>0.05; Rev-Control males: F(9,210)=5.112, p<0.05; Acq-VAChTcKO females: p>0.05; Rev-VAChTcKO females: p>0.05; Acq-VAChTcKO males: p>0.05; Rev-VAChTcKO males: p>0.05). (h) No sex differences and across genotypes were observed in the total time the reward magazine (RM) was visited within sessions (p>0.05). A total of N=24 (n=13♂, n=11♀) control littermate (VAChT$^{flox/flox}$) and N=25 (n=11♂, n=14♀) VAChTcKO mice were used during acquisition and reversal sessions. Post-hoc Tukey's test: ***p<0.0001, **p<0.001, *p<0.05. No adjustments were made for multiple comparison analyses. Data are presented as the mean ± SEM. Source data are provided as a Source Data file.

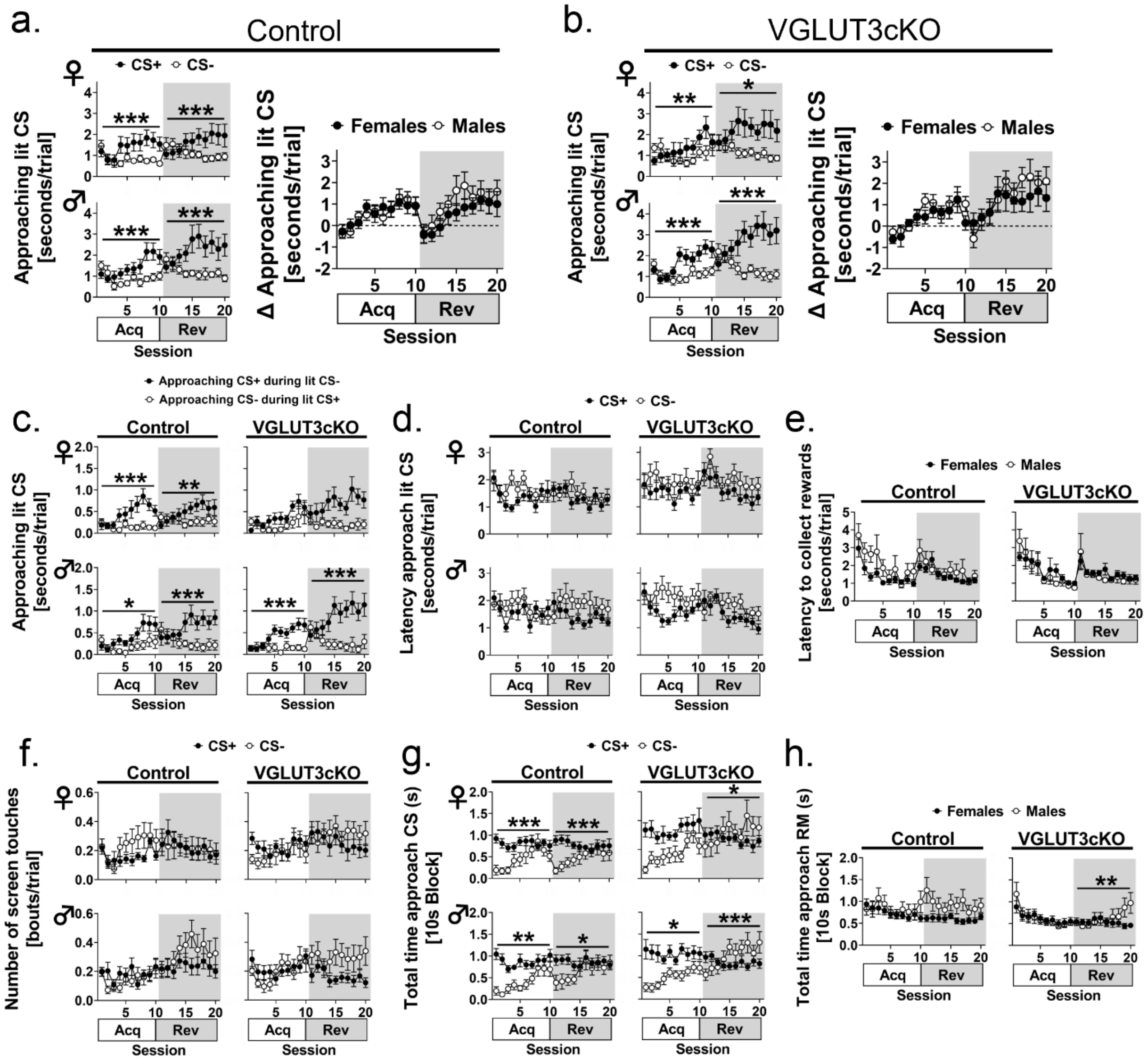

Supplementary Figure 4: VGLUT3cKO mice approach reward-predicting stimuli.

(a) (left panels) Female and male control littermate mice similarly spent more time approaching the CS+ (filled circles) than the CS- (blank circles) during acquisition (Acq, S1→S10) and reversal (Rev, S11→S20) training sessions (two-way RM-ANOVA SessionXCS interaction, Acq-females: $F(9,198)=3.926$, $p=0.0001$; Rev-females: $F(9,198)=5.263$, $p<0.0001$; Acq-males: $F(9,198)=3.572$, $p=0.0004$; Rev-males: $F(9,198)=5.815$, $p<0.0001$). (right panel) The relative time (Δ) mice spent visiting the CS revealed that females (filled circles) and males (blank circles) similarly approached

more the CS+ than the CS- ($p>0.05$). (b) (left panels) Female and male VGLUT3cKO mice also approached more the CS+ than the CS- across sessions (Mixed-effects Model SessionXCS interaction, Acq-females: $F(9,178)=3.042$, $p<0.001$; Rev-females: $F(9,179)=2.129$, $p<0.01$; Acq-males: $F(9,198)=4.988$, $p<0.0001$; Rev-males: $F(9,198)=8.217$, $p<0.0001$). (right panel) No sex differences were observed when compared the relative time (Δ) mice approached the CS across sessions ($p>0.05$). (c) During CS+ presentation (blank circles), female and male mice from both genotypes rarely explored the CS-. Instead, mice visited the CS+ screen when CS- was presented (filled circles) (Mixed-effects Model SessionXCS interaction, Acq-Control females: $F(9,198)=4.987$, $p<0.0001$; Rev-Control females: $F(9,198)=3.226$, $p<0.001$; Acq-Control males: $F(9,198)=2.322$, $p<0.01$; Rev-Control males: $F(9,198)=4.816$, $p<0.0001$; Acq-VGLUT3cKO females: $p>0.05$; Rev-VGLUT3cKO females: $p>0.05$; Acq-VGLUT3cKO males: $F(9,198)=6.000$, $p<0.0001$; Rev-VGLUT3cKO males: $F(9,198)=6.017$, $p<0.0001$). (d) No sex or genotype differences were observed in the latency time mice spent to approach the CS ($p>0.05$). (e) Latency time to collect rewards was similar across sex and between genotypes ($p>0.05$). (f) No sex differences at touching both CS+/CS- screens during presentation were observed in both control littermate and VGLUT3cKO mice ($p>0.05$). (g) Recording the total time mice approached both CS screens within the session revealed more visits the CS+ than the CS- (Mixed-effects Model SessionXCS interaction, Acq-Control females: $F(9,198)=4.482$, $p<0.0001$; Rev-Control females: $F(9,197)=3.835$, $p<0.0001$; Acq-Control males: $F(9,198)=3.000$, $p<0.001$; Rev-Control males: $F(9,198)=2.473$, $p<0.01$; Acq-VGLUT3cKO females: $p>0.05$; Rev-VGLUT3cKO females: $F(9,179)=2.240$, $p<0.01$; Acq-VGLUT3cKO males: $F(9,198)=2.027$, $p<0.01$; Rev-VGLUT3cKO males: $F(9,198)=4.385$, $p<0.0001$). No differences between sex and across genotypes were observed ($p>0.05$). (h) No sex differences between genotypes were observed in the total time the reward magazine (RM) was visited across sessions ($p>0.05$). We found that male VGLUT3cKO mice visited more the RM during reversal training sessions (one-way ANOVA, $F(9,189)=3.176$, $p<0.001$), but not during acquisition sessions ($p>0.05$). A total of N=24 (n=12♂, n=12♀) control littermate (VGLUT3$^{flox/flox}$) and N=23 (n=12♂, n=11♀) VGLUT3cKO mice were used during acquisition and reversal sessions. Post-hoc

Tukey's test: ***p<0.0001, **p<0.001, *p<0.05. No adjustments were made for multiple comparison analyses. Data are presented as the mean ± SEM. Source data are provided as a Source Data file.

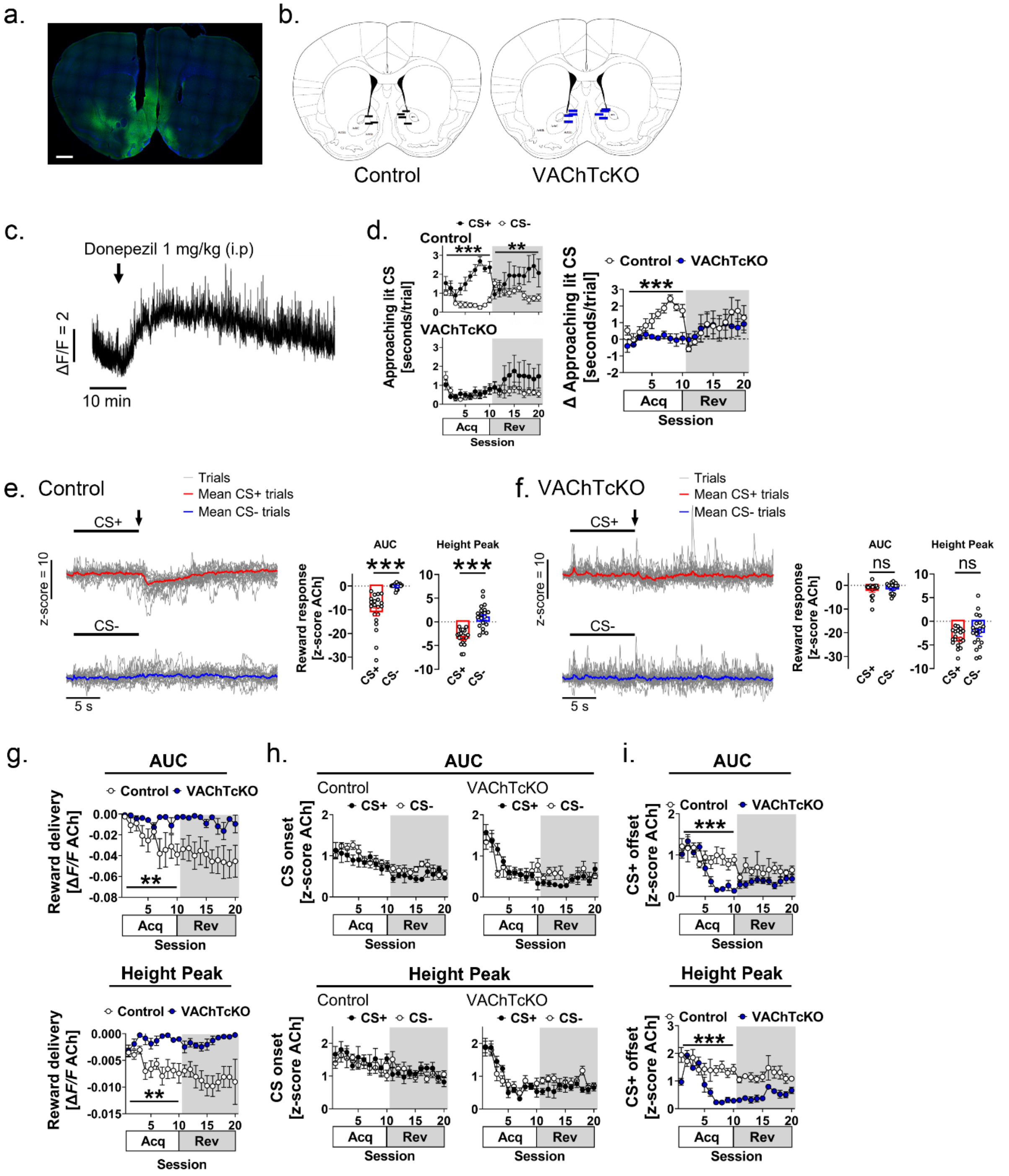

Supplementary Figure 5: Acetylcholine dynamics in control littermate and VAChTcKO mice performing the Autoshaping task.

(a) Representative brain section showing GFP immunoreactivity (ACh3.0, green) around the probe track within the nucleus accumbens. Nuclei were stained with Hoechst (blue). Scale bar–500μm. (b) Schematic brain sections illustrating fibre optic lesions

above the nucleus accumbens of control littermate (black bars) and VAChTcKO mice (blue bars). (c) Representative ACh dynamics (ΔF/F) from a mouse expressing ACh3.0 within the nucleus accumbens. After 5 min baseline recordings, mice received a systemic injection of donepezil (arrow bar, 1mg.kg-1, i.p.) and signal recorded for 1h post-injection. (d) (left panels) In contrast to control littermate mice, VAChTcKOs did not spend more time approaching the CS+ across acquisition (Acq, S1→S10) and reversal (Rev, S11→S20) sessions (two-way RM-ANOVA SessionXCS interaction, Acq-Control: $F(9,108)=6.398$, $p<0.0001$; Rev-Control: $F(9,108)=3.027$, $p=0.0029$; Acq-VAChTcKO: $p>0.05$; Rev-VAChTcKO: $p>0.05$). Consistently, the relative time (Δ) mice approached the CS showed that VAChTcKOs (blue circles) were significantly impaired when compared to control littermate mice (blank circles) during acquisition but reversal sessions (two-way RM-ANOVA SessionXGenotype interaction, Acq: $F(9,117)=3.761$, $p=0.0003$; Rev: $p>0.05$). (e) (left) Representative ACh dynamics (z-score) from individual trials (gray traces) and trial average of CS+ (red) and CS- (blue) contingencies from a control littermate mouse at S10. Bar represents CS presentation (10s), arrow bar reward delivery. (right) Area under the curve (AUC) and height peak of individual trials (blank circles) and trial average (CS+, red; CS-, blue) after CS offset. Both AUC and height peaks (two-tailed t test, AUC: $t(19)=6.076$, $p<0.0001$; height peak: $t(19)=6.880$, $p<0.0001$) events were significantly larger during CS+ than CS- contingencies. (f) (left) Representative ACh dynamics (z-score) from individual trials (gray traces) and trial average of CS+ (red) and CS- (blue) from a VAChTcKO mouse at S10. (right) No differences in the amplitude of ACh events after CS+ or CS- offset were observed when comparing the AUC ($p>0.05$) or height peak ($p>0.05$) of individual trials. (g) The amplitude of the ACh response (top, AUC ΔF/F; bottom, height peak ΔF/F) during the reward delivery was significantly impaired in VAChTcKO mice when compared to control littermate mice (two-way RM-ANOVA SessionXCS interaction, Acq-AUC: $F(9,117)=3.234$, $p=0.0015$; Rev-AUC: $p>0.05$; Acq-height peak: $F(9,117)=3.275$, $p=0.0014$; Rev-height peak: $p>0.05$). (h) (top-panels) Analysis of AUC and (bottom-panels) height peaks of ACh events during CS+ (filled circles) and CS- (blank circles) onset in control littermate and VAChTcKO mice across sessions. No differences in the ACh dynamics were observed across contingencies or between genotypes ($p>0.05$). (i)

(top) AUC or (bottom) height peak of ACh events during CS+ offset in control littermate (filled circles) and VAChTcKO mice (blank circles) across sessions. During acquisition sessions, both AUC and height peak analysis revealed that the ACh response in VAChTcKOs was significantly reduced as compared to control littermate mice (two-way RM-ANOVA Genotype factor, AUC: $F(1,13)=13.23$, $p=0.003$; Height peak: $F(1,13)=20.90$, $p=0.0005$), whereas during reversal sessions, the height peak but AUC analysis demonstrated that ACh signal response was reduced in VAChTcKOs (two-way RM-ANOVA Genotype factor, AUC: $p>0.05$; Height peak: $F(1,13)=62.33$, $p<0.0001$). A total of N=7 (n=4♂, n=3♀) control littermate ($VAChT^{flox/flox}$) and N=8 (n=4♂, n=4♀) VAChTcKO mice were used during acquisition and reversal sessions. Post-hoc Tukey's test: ***$p<0.0001$, **$p<0.001$, *$p<0.05$. No adjustments were made for multiple comparison analyses. Data are presented as the mean ± SEM. Source data are provided as a Source Data file.

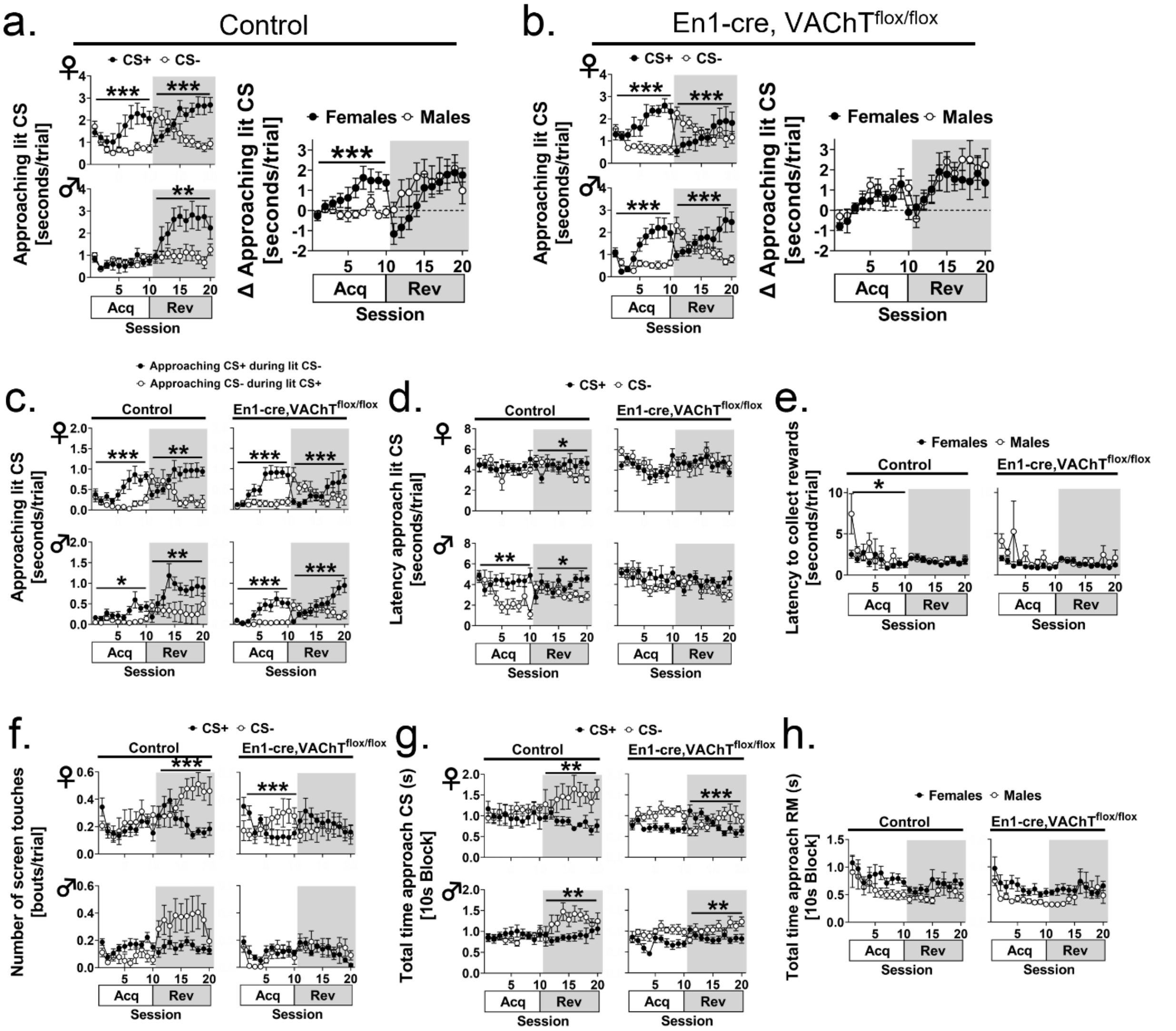

Supplementary Figure 6: Impaired acetylcholine release from pedunculopontine/laterodorsal tegmental projecting neurons does not affect approaches towards reward-predicting cues.

(a) (left panels) Female and male control littermate (VAChT$^{flox/flox}$) mice spent more time approaching the CS+ (filled circles) than CS- (blank circles) during acquisition (Acq, S1→S10) and reversal (Rev, S11→S20) sessions (two-way RM-ANOVA SessionXCS interaction, Acq-females: $F(9,144)=4.779$, $p<0.0001$; Rev-females: $F(9,144)=11.59$, $p<0.0001$; Acq-males: $p>0.05$; Rev-males: $F(9,144)=2.698$, $p=0.0063$). (right panel) The relative time (Δ) mice spent visiting both CS revealed that during acquisition sessions,

males (blank circles) did not discriminate between CS+ and CS- as compared to females (filled circles) (two-way RM-ANOVA SessionXSex, acquisition: $F(9,144)=3.780$, $p=0.0003$; reversal: $p>0.05$). (b) (left panels) Female and male En1-Cre,VAChT$^{flox/flox}$ mice also approached more the CS+ than CS- during training sessions (two-way RM-ANOVA SessionXCS interaction, Acq-females: $F(9,126)=9.727$, $p<0.0001$; Rev-females: $F(9,126)=8.490$, $p<0.0001$; Acq-males: $F(9,144)=8.479$, $p<0.0001$; Rev-males: $F(9,144)=8.322$, $p<0.0001$). (right panel) No sex differences were observed when compared the relative (Δ) time to approach the CS across sessions ($p>0.05$). (c) During CS- stimulus presentation (filled circles), female and male mice visited the CS+ screen. In contrast, almost no visits were recorded towards the CS- screen when the CS+ was displayed (blank circles) (two-way RM-ANOVA SessionXCS interaction, Acq-control females: $F(9,144)=3.886$, $p=0.0002$; Rev-control females: $F(9,144)=7.562$, $p<0.0001$; Acq-control males: $F(9,144)=2.218$, $p=0.0240$; Rev-control males: $F(9,144)=3.091$, $p=0.0020$; Acq-En1-Cre,VAChT$^{flox/flox}$ females: $F(9,126)=6.050$, $p<0.0001$; Rev-En1-Cre,VAChT$^{flox/flox}$ females: $F(9,144)=7.686$, $p<0.0001$; Acq-En1-Cre,VAChT$^{flox/flox}$ males: $F(9,144)=5.079$, $p<0.0001$; Rev-En1-Cre,VAChT$^{flox/flox}$ males: $F(9,144)=5.565$, $p<0.0001$). (d) Although no sex or genotype differences were observed between mice ($p>0.05$), the latency time to approach the CS- was longer in male control mice (two-way RM-ANOVA SessionXCS interaction, Acq-control females: $p>0.05$; Rev-control females: $F(9,144)=2.188$, $p=0.0261$; Acq-control males: $F(9,144)=2.874$, $p=0.0038$; Rev-control males: $F(9,144)=2.094$, $p=0.0337$; Acq-En1-Cre,VAChT$^{flox/flox}$ females: $p>0.05$; Rev-En1-Cre,VAChT$^{flox/flox}$ females: $p>0.05$; Acq-En1-Cre,VAChT$^{flox/flox}$ males: $p>0.05$; Rev-En1-Cre,VAChT$^{flox/flox}$ males: $p>0.05$). (e) Latency time to collect rewards was similar between genotypes ($p>0.05$). A longer latency time was observed during acquisition sessions in male control littermate mice (two-way RM-ANOVA SessionXSex, Acq-control: $F(9,144)=2.079$, $p=0.0351$; Rev-control: $p>0.05$; Acq-En1-Cre,VAChT$^{flox/flox}$: $p>0.05$; Rev-En1-Cre,VAChT$^{flox/flox}$: $p>0.05$). (f) No sex differences at touching both CS stimuli during presentation were observed in both control littermate and En1-Cre,VAChT$^{flox/flox}$ mice ($p>0.05$). Furthermore, a bias towards the CS- presentation was observed during reversal sessions in control mice (two-way RM-ANOVA SessionXCS interaction, Acq-females: $p>0.05$; Rev-females: $F(9,144)=6.112$, $p<0.001$; Acq-males:

$p>0.05$; Rev-males: $p>0.05$), but during acquisition sessions in female En1-cre,VAChT$^{flox/flox}$ mice (two-way RM-ANOVA SessionXCS interaction, Acq-females: $F(9,126)=3.933$, $p=0.0002$; Rev-females: $p>0.05$; Acq-males: $p>0.05$; Rev-males: $p>0.05$). (g) No differences between sex and across genotypes were observed when recording the total time mice approached both CS screens within the session regardless of trial contingency ($p>0.05$). However, both male and female mice showed a preference to visit the CS- screen during reversal sessions (two-way RM-ANOVA SessionXCS interaction, Acq-control females: $p>0.05$; Rev-control females: $F(9,144)=3.128$, $p=0.0018$; Acq-control males: $p>0.05$; Rev-control males: $F(9,144)=2.627$, $p=0.0077$; Acq-En1-Cre,VAChT$^{flox/flox}$ females: $p>0.05$; Rev-En1-cre,VAChT$^{flox/flox}$ females: $F(9,144)=9.248$, $p<0.0001$; Acq-En1-cre,VAChT$^{flox/flox}$ males: $p>0.05$; Rev-En1-cre,VAChT$^{flox/flox}$ males: $F(9,144)=2.919$, $p=0.0033$). (h) No sex differences and across genotypes were observed in the total time the reward magazine (RM) was visited within sessions ($p>0.05$). A total of N=18 (n=9♂, n=9♀) control littermate (VAChT$^{flox/flox}$) and N=17 (n=9♂, n=8♀) En1-Cre;VAChT$^{flox/flox}$ mice were used during acquisition and reversal sessions. Post-hoc Tukey's test: ***$p<0.0001$, **$p=0.001$, *$p<0.05$. No adjustments were made for multiple comparison analyses. Data are presented as the mean ± SEM. Source data are provided as a Source Data file.

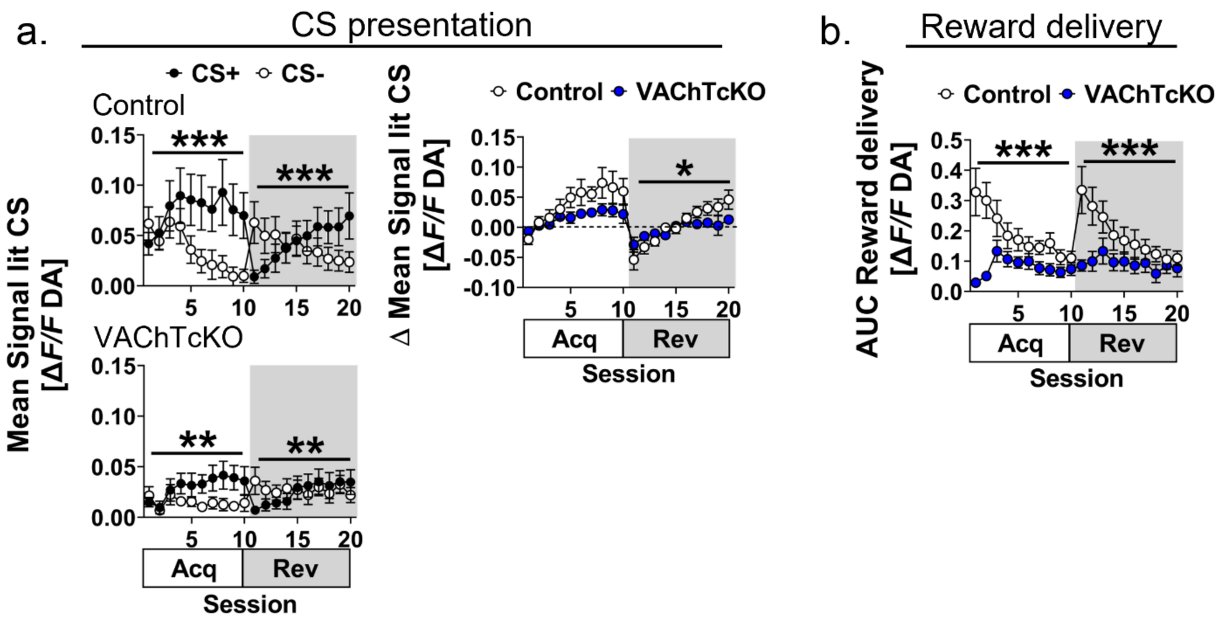

Supplementary Figure 7: Amplitude of dopamine dynamics are reduced in VAChTcKO mice

(a) (left panels) Mean DA dynamics (ΔF/F) in control littermate mice (VAChT$^{flox/flox}$) were significantly larger during CS+ than CS- in both acquisition (Acq) and reversal (Rev) sessions (two-way RM-ANOVA SessionXCS interaction, Acq: $F(9,126)=5.061$, $p<0.0001$; Rev: $F(9,126)=13.28$, $p<0.0001$). Moreover, the DA dynamics were also larger in CS+ than CS- in VAChTcKO mice across sessions (two-way RM-ANOVA SessionXCS, Acq: $F(9,108)=2.765$, $p=0.006$; Rev: $F(9,108)=3.162$, $p=0.002$). (right panels) Compared to control mice (blank circles), the relative increase of DA signal (Δ) was significantly lower toward the CS+ in VAChTcKO mice (blue circles) (two-way RM-ANOVA SessionXCS interaction, Acq: $p>0.05$; Rev: $F(9,117)=2.527$, $p=0.011$). (b) Compared to control littermate mice (blank circles), the amplitude of the DA response during reward delivery (AUC, ΔF/F) was blunted in VAChTcKO mice (blues circles) across sessions (two-way RM-ANOVA SessionXCS interaction, Acq: $F(9,117)=6.278$, $p<0.0001$; Rev: $F(9,117)=4.390$, $p<0.0001$). A total of N=8 (n=4♂, n=3♀) control littermate mice and N=7 (n=3♂, n=4♀) VAChTcKO mice were used. Post-hoc Tukey's test: ***$p<0.0001$, **$p<0.01$, *$p<0.05$. No adjustments were made for multiple comparison analyses. Data are presented as the mean ± SEM. Source data are provided as a Source Data file.

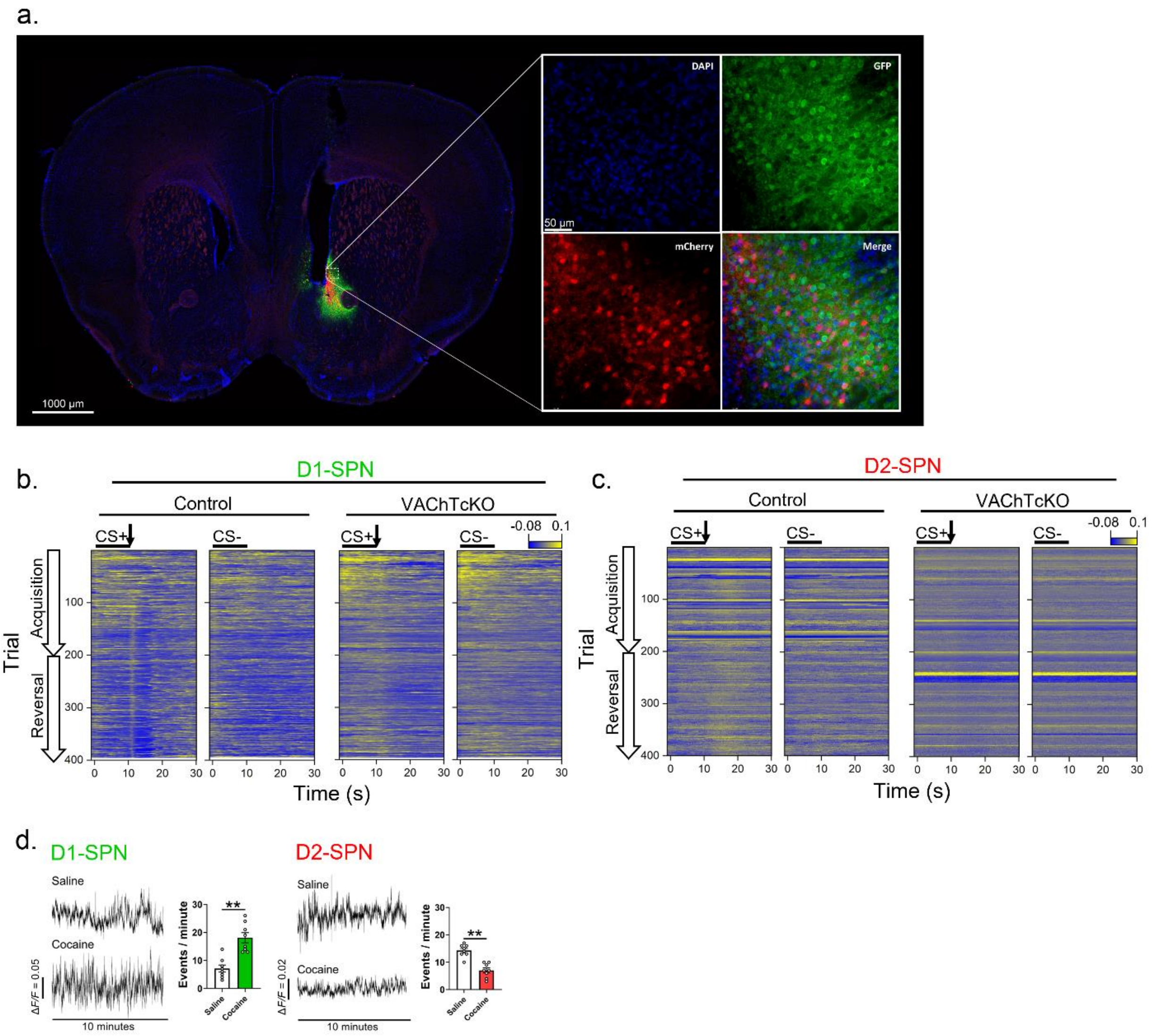

Supplementary Figure 8: Acetylcholine release from cholinergic interneurons regulates the concurrent calcium activity of the direct and indirect spiny projecting neurons in nucleus accumbens.

(a) Representative coronal brain section from a control (D2-Cre) mouse showing probe track and immunoreactivity to GFP (GCaMP6s, green) in putative D1-SPNs and mCherry (jRCaPM1a, red) in putative D2-SPNs. The boxed area in the left is enlarged on the right to show the numerous neurons expressing either GFP or mCherry. Nuclei were stained with DAPI (blue). Immunostaining was reproduced in a total of N=9 control (D2-Cre) and N=10 VAChTcKO mice. Scale bar–1000µm. (b) Trial by trial calcium

dynamics (ΔF/F) from putative D1-SPNs within the nucleus accumbens of a representative control and VAChTcKO mouse performing the Autoshaping task (acquisition, trials 1→200; reversal, trials 201→400). The bar indicates the presentation of the CS+ or CS- (10s). The arrow bar indicates the time of reward delivery. (c) trial by trial calcium dynamics (ΔF/F) from putative D2-SPNs within the nucleus accumbens of a representative control and VAChTcKO mouse performing the Autoshaping task. The bar indicates the presentation of the CS+ or CS- (10s). The arrow bar indicates the time when the reward was delivered. (d) Representative calcium dynamics (ΔF/F) of D1-SPNs and D2-SPNs within the nucleus accumbens of control mice (N=8). Homecage calcium dynamics demonstrated that after a saline injection (i.p.), more events were observed in D2-SPNs than in D1-SPNs (two tailed t test, $t(14)=4.781$, $p=0.0003$). Then, mice received a single cocaine injection (10 $mg.kg^{-1}$, i.p.) and compared to their saline baseline levels. The number of events significantly increased in D1-SPNs (two-tailed t test, $t(7)=5.010$, **$p=0.0015$) but decreased in D2-SPN ($t(7)=5.093$, **$p=0.0014$). Source data are provided as a Source Data file.
