# Supplementary material for: Continuous cholinergic-dopaminergic updating in the nucleus accumbens underlies approaches to reward-predicting cues
Source: Nat Commun. 2022 Dec 24;13:7924. doi: 10.1038/s41467-022-35601-x (PMC9789106; doi:10.1038/s41467-022-35601-x)
Supplement: Supplementary file 3 — Reporting Summary [file 41467_2022_35601_MOESM3_ESM.pdf]

Corresponding author(s): Miguel Skirzewski, Marco A.M. Prado, Timothy J. Bussey

Last updated by author(s): Nov 21, 2022

Reporting Summary

Nature Portfolio wishes to improve the reproducibility of the work that we publish. This form provides structure for consistency and transparency in reporting. For further information on Nature Portfolio policies, see our Editorial Policies and the Editorial Policy Checklist.

Statistics

For all statistical analyses, confirm that the following items are present in the figure legend, table legend, main text, or Methods section.

n/a	Confirmed	
☐	☒	The exact sample size (*n*) for each experimental group/condition, given as a discrete number and unit of measurement
☐	☒	A statement on whether measurements were taken from distinct samples or whether the same sample was measured repeatedly
☐	☒	The statistical test(s) used AND whether they are one- or two-sided<br>*Only common tests should be described solely by name; describe more complex techniques in the Methods section.*
☐	☒	A description of all covariates tested
☐	☒	A description of any assumptions or corrections, such as tests of normality and adjustment for multiple comparisons
☐	☒	A full description of the statistical parameters including central tendency (e.g. means) or other basic estimates (e.g. regression coefficient) AND variation (e.g. standard deviation) or associated estimates of uncertainty (e.g. confidence intervals)
☐	☒	For null hypothesis testing, the test statistic (e.g. *F*, *t*, *r*) with confidence intervals, effect sizes, degrees of freedom and *P* value noted<br>*Give P values as exact values whenever suitable.*
☒	☐	For Bayesian analysis, information on the choice of priors and Markov chain Monte Carlo settings
☒	☐	For hierarchical and complex designs, identification of the appropriate level for tests and full reporting of outcomes
☐	☒	Estimates of effect sizes (e.g. Cohen's *d*, Pearson's *r*), indicating how they were calculated

Our web collection on statistics for biologists contains articles on many of the points above.

Software and code

Policy information about availability of computer code

Data collection	Behavioural data was collected using the automated Bussey-Saksida Mouse Touchscreen Systems Model 80614-20 (Lafayette Instruments). Schedule design, control of the apparatus via Whisker control system (v-4.7) (Cambridge University Technical Services), and data collection used ABET II Video Touch software (v-21.02.26, Campdem Instruments).<br>Western blot images were visualized using the Image Lab Touch software (v-2.2.0.08) from the 685 ChemiDoc MP Imaging System (BioRad).<br>Microscopy images were collected using the Leica Application Suite X software (v-3.7.5.24914) from the Leica DM6B Thunder microscope (Leica Microsystems Inc).<br>Fibre photometry fluorescence signal was collected using Doric Studio (v-5.2.2.3, Doric Lenses).<br>All behavioural and fibre photometry raw fluorescence data is freely available at Mousebytes (https://mousebytes.ca/home).
Data analysis	Behavioral data was extracted using the ABET II Video Touch software (v-21.02.26) (Campdem Instruments), and analyzed with Graph Pad Prism (v-9.3.1), and an open source Phyton-based code (v-3.7) for public open-access repository and visualization (mousebytes.ca).<br>Demodulated fiber photometry data was extracted and analyzed using an open source Phyton-based code (v-3.7) for public open-access repository and visualization (https://mousebytes.ca/home), Graphpad Prism (v-9.3.1), and OriginPro 2021 64-bit (v-9.8).<br>Code availability for fibre photometry data can be accessed from the Mousebyte complementary repository (https://mousebytes.ca/comp-edit?repolinkguid=ccf27660-6442-4c90-a14c-cdbc663a7b72)<br>Microscopy images were analyzed using ImageJ 64-bit (v-1.8) and Graphpad Prism (v-9.3.1).

For manuscripts utilizing custom algorithms or software that are central to the research but not yet described in published literature, software must be made available to editors and reviewers. We strongly encourage code deposition in a community repository (e.g. GitHub). See the Nature Portfolio guidelines for submitting code & software for further information.

Data

Policy information about availability of data

All manuscripts must include a data availability statement. This statement should provide the following information, where applicable:

- Accession codes, unique identifiers, or web links for publicly available datasets
- A description of any restrictions on data availability
- For clinical datasets or third party data, please ensure that the statement adheres to our policy

The data supporting these findings can be visualized and are freely accesible at Mousebytes (https://mousebytes.ca/home). The code availability and data can be accessed through the complementary Mousebytes repository (https://mousebytes.ca/comp-edit?repolinkguid=ccf27660-6442-4c90-a14c-cdbc663a7b72).

Human research participants

Policy information about studies involving human research participants and Sex and Gender in Research.

Reporting on sex and gender	N/A
Population characteristics	N/A
Recruitment	N/A
Ethics oversight	N/A

Note that full information on the approval of the study protocol must also be provided in the manuscript.

Field-specific reporting

Please select the one below that is the best fit for your research. If you are not sure, read the appropriate sections before making your selection.

☒ Life sciences ☐ Behavioural & social sciences ☐ Ecological, evolutionary & environmental sciences

For a reference copy of the document with all sections, see nature.com/documents/nr-reporting-summary-flat.pdf

Life sciences study design

All studies must disclose on these points even when the disclosure is negative.

Sample size	The sample sizes used in our study are about the same or exceed those estimated by power analysis (power = 0.9, α = 0.05).
Data exclusions	For fiber photometry experiments, only animals with incorrect probe placement were excluded from analysis. Otherwise, no animals were excluded from analysis.
Replication	Experiments were performed in at least three independent cohort of mice per experimental group. All replicates were successful.
Randomization	Similar number of male and female mice were equally distributed between experimental groups.
Blinding	All cell counting, in situ hybridization, and immunohistochemistry, experiments were performed by an experimenter blind to the experimental conditions. In contrast, behavioural and combined fibre photometry data were collected using the automated touchscreen systems. Therefore, data collection and analysis were not feasible to be performed blind to the experimental conditions. Nevertheless, the automated touchscreens allow for unbiased data collection and analysis as all the fibre photometry recordings and behavioural contingencies are automatically generated by the system.

Reporting for specific materials, systems and methods

We require information from authors about some types of materials, experimental systems and methods used in many studies. Here, indicate whether each material, system or method listed is relevant to your study. If you are not sure if a list item applies to your research, read the appropriate section before selecting a response.

Materials & experimental systems

n/a	Involved in the study
☐	☒ Antibodies
☒	☐ Eukaryotic cell lines
☒	☐ Palaeontology and archaeology
☐	☒ Animals and other organisms
☒	☐ Clinical data
☒	☐ Dual use research of concern

Methods

n/a	Involved in the study
☒	☐ ChIP-seq
☒	☐ Flow cytometry
☒	☐ MRI-based neuroimaging

Antibodies

Antibodies used

Rabbit anti-VAChT. Supplier: Synaptic Systems, Catalog #: 139103, Clone name: N/A, Lot #: 4-53
Mouse anti-beta actin. Supplier: Sigma-Aldrich, Catalog #: A3854, Clone name: AC-15, Lot #: 047M4792V
Chicken anti-GFP. Supplier: Abcam, Catalog#: ab13970, Clone name: N/A, Lot#: GR3190550-17
Rabbit anti-mCherry. Supplier: Abcam, Catalog#: ab167453, Clone name: N/A, Lot#: GR3265215-3
Rabbit anti-Synaptophysin. Supplier: Cell Signaling Technology, Catalog#: 5461S, Clone name: N/A, Lot#: 3
Goat anti-Rabbit HRP. Supplier: BioRad, Catalog#: 170-6515, Clone name: N/A, Lot#: 64461887
Goat anti-Chicken Alexa Fluor 488. Supplier: Thermo Fisher, Catalog#: A11039, Clone name: N/A, Lot#: 1812246
Goat anti-Rabbit Alexa Fluor 633. Supplier: Thermo Fisher, Catalog#: A21070, Clone name: N/A, Lot#1668691

Validation

The information provided below is as described by the corresponding manufacturers.
Rabbit anti-VAChT. VAChT. 139 103-WB, dilution: 1:1000, sample: enriched synaptic vesicles from rat brain (LP2), Detection: AP-staining, reacts with: human (Q16572), rat (Q62666), mouse (O35304), pig, zebrafish. Other species not tested yet. Specific for VAChT K.O. PubMed: 24027290, https://sysy.com/product/139103.
Mouse anti-beta actin. Species reactivity: Pig, Hirudo medicinalis, bovine, rat, canine, feline, human, rabbit, carp, mouse, guinea pig, chicken, sheep. Monoclonal mouse anti-actin antibody was used as a loading control for western blot analysis of immunoprecipitated proteins from rat dorsal root ganglion co-cultures. Monoclonal mouse anti-actin was used as a loading control for western blot analysis of rat liver protein lysates. Mouse monoclonal anti-β-actin-peroxidase antibody can be used as a primary antibody for western blot assays. Monoclonal Anti-ß-Actin-Peroxidase antibody has been used in western blot analysis and chemiluminescent detection. From: https://www.sigmaaldrich.com/CA/en/product/sigma/a3854.
Chicken anti-GFP. This antibody has been tested in the following applications: WB, ICC/IF. Reacts with: Species independent. From: https://www.abcam.com/gfp-antibody-ab13970.html.
Rabbit anti-mCherry. This antibody has been tested in the following applications: WB, ICC/IF. Reacts with: Species independent. From: https://www.abcam.com/mcherry-antibody-ab167453.html.
Rabbit anti-Synaptophysin. Synaptophysin (D35E4) Rabbit mAb detects endogenous levels of total synaptophysin protein. Species Reactivity: Human, Mouse, Rat. Application: western blotting, immunoprecipitation. From: https://www.cellsignal.com/products/primary-antibodies/synaptophysin-d35e4-rabbit-mab/5461.

Animals and other research organisms

Policy information about studies involving animals; ARRIVE guidelines recommended for reporting animal research, and Sex and Gender in Research

Laboratory animals

This study used adult 3-7 month old mice. Animals were housed in groups of two to four per cage at 22–23°C , 40-60% humidity, with a 12:12h reverse light-dark cycle. Food and water were provided ad libitum until behavioural testing, at which point mice were mildly food restricted (90–95% of their original body weight) to increase their motivation to perform a behavioural task. Experiments were performed during the dark cycle (between 9 a.m. and 6 p.m.).
Animals used:
C57BL/6j
VAChTflox/flox
VGLUT3flox/flox
D2-cre [Tg(Drd2-cre) 44Gsat; GENSAT obtained from MMRRC B6/129/ Swiss/FVB mixed background]
Engrailed-1 (En1)-Cre mice [Jax# 007916; 129S1/SvImJ mixed background]
D2-Cre;VAChTflox/flox
D2-Cre;VGLUT3flox/flox
En1-Cre,VAChTflox/flox

Wild animals

This study did not involve wild animals.

Reporting on sex

This study used male and female mice.
Male N=122
Female N=120

Field-collected samples

This study did not use samples collected from the field.

Ethics oversight

The use and care of the animals was conducted in agreement with the Canadian Council of Animal Care guidelines and the animal protocols approved by the Animal Care and Veterinary Services from Western University (protocols #2020-162, 2020-163).

Note that full information on the approval of the study protocol must also be provided in the manuscript.
